# Compression Strength Estimation of Corrugated Board Boxes for a Reduction in Sidewall Surface Cutouts—Experimental and Numerical Approaches

**DOI:** 10.3390/ma16020597

**Published:** 2023-01-07

**Authors:** Lajos Fehér, Renáta Pidl, Péter Böröcz

**Affiliations:** 1 Department of Applied Mechanics, Széchenyi István University, Egyetem tér 1, 9026 Győr, Hungary; 2 Department of Logistics and Forwarding, Széchenyi István University, Egyetem tér 1, 9026 Győr, Hungary

**Keywords:** paperboard packaging, box compression test, package design, McKee formula, cutout

## Abstract

Corrugated cardboard boxes are generally used in modern supply chains for the handling, storage, and distribution of numerous goods. These packages require suitable strength to maintain adequate protection within the package; however, the presence and configuration of any cutouts on the sidewalls significantly influence the packaging costs and secondary paperboard waste. This study aims to evaluate the performance of CCBs by considering the influence of different cutout configurations of sidewalls. The compression strength of various B-flute CCB dimensions (200 mm, 300 mm, 400 mm, 500 m, and 600 mm in length, with the same width and height of 300 mm), each for five cutout areas (0%, 4%, 16%, 36%, and 64%) were experimentally observed, and the results were compared with the McKee formula for estimation. The boxes with cutout areas of 0%, 4%, 16%, 36%, and 64% showed a linear decreasing tendency in compression force. A linear relationship was found between compression strength and an increase in cutout sizes. Packages with 0% and 4% cutouts did not show significant differences in compression strength (*p* < 0.05). Furthermore, this study shows a possible way to modify the McKee estimation for such boxes after obtaining empirical test data since the McKee formula works with a relatively high error rate on corrugated cardboard boxes with sidewall cutouts. Utilizing the numerical and experimental results, a favorable estimation map can be drawn up for packaging engineers to better manage material use and waste. The results of the study showed that the McKee formula does not appropriately estimate the box compression strength for various cutout sizes in itself.

## 1. Introduction

Most of the goods transported use various transport packaging, including the most often used paperboard packaging. This is the leading material to ensure the necessary protection and logistical aims, such as stacking, handling, and forming unit loads during distribution, due to its load-bearing capacity and other beneficial properties [1,2]. The main advantages of paper-based packaging are the reliable protection of products, in addition to relatively low packaging costs, recyclability, and biodegradability [3,4]. Stacking packages on top of each other can cause damage to the packaged product, so before doing this, it is necessary to find an appropriate estimation method or to perform a series of tests to determine the mechanical strength of various constructions of cardboard boxes [4,5,6]. The situation is more complex if there are some special requirements for the box, such as hand holes, ventilation holes, and openings, respectively [7,8,9,10,11]. Additional considerations involving corrugated cardboard boxes (CCBs) are optimal packaging material costs and sustainable development strategies within their product-packaging range [12]. Basically, the cost of CCB packaging depends on the number of layers and paper/cardboard quality, which are in relation to the mass used [13]. The latter, of course, impacts the cost of packaging solutions and the final mass for destruction or recycling after use. Therefore, it is in the interest of the actors in the industry to find the box with optimal mechanical properties, which, on the one hand, adequately ensures product protection and, on the other hand, leads to acceptable packaging costs and waste savings.

Corrugated cardboard is made of odd layers, usually three or five. In the case of three-layered corrugated cardboard, the corrugated layer is placed between the inner and outer flat layers, while five-layered cardboard has two corrugated layers. There are also several types of corrugated layers according to flute height and flute length. The individual layers are bonded together by glue. In the paper industry, the highest flute is denoted by A, followed by flute C, and the lowest normal flute is B. There are so-called micro-flutes with the letters E and F (FEFCO, European Federation of Corrugated Board Manufacturers) [14].

The optimal package design of corrugated boxes for packaging engineers is a major challenge [15]. When trying to ensure product-packaging integrity, the situation is further complicated by environmental conditions that can affect the mechanical behavior of corrugated board, including temperature, relative humidity changes, perforations, prints, etc. [16,17], which can decrease the resilience of the integrity of the packaged product to damages [8,9,10,11,18]. However, the most critical issue of compression strength for CCBs is the presence of any openings, handling, or ventilation holes on the walls, and their dimension, orientation, shape, and number [8,19]. 

One possible way to determine the strength of corrugated boxes is to perform a series of tests in laboratory conditions following standard protocols. In the paper packaging industry, these tests cover the compression, bending, and bursting of corrugated boards. However, the most important and practical tests are the box compression test (BCT) and the edge crush test (ECT). The latter can give information to use as input in an analytical formula for predicting compressive strength. The well-known semi-empirical formula to determine the possible stacking load is McKee’s equation [18,20]. The McKee formula is a simple, practical application using parameters of paper, board, and boxes with an arbitrarily chosen constant. The disadvantage of the method is that the formula is applicable to relatively typical box containers without modification of holes, cutouts, and so forth.

In the last 50 years, many researchers have tried to extend the applicability of McKee’s formula and presented different approaches. There is a method by Kellicutt and Landt [21] that developed a model for compressive load sizing based on the principle of annular compressive strength. Beldie et al. presented a study in 2001 that modeled the mechanical behavior of corrugated cardboard packages subjected to static compressive loading [22]. Biancolini and Brutti [23] presented a numerical model for splitting the properties of corrugated cardboard boxes with strength calculations. Allerby et al. [24] presented a study in 1985 in which they modified the constants and exponents of McKee’s formula. In 1987, Schrampfer et al. extended the applicability of the McKee relationship to a wider range of cutting methods and equipment with a combined board-edge crush technology [25]. Furthermore, the McKee constant was later analyzed in more sophisticated way in complicated cases by Garbowski et al. [26]. There are studies in which the authors have shown that additional tests are required and, therefore, an updated formulation should be used, which was recently modified by Aviles et al. [27] and later by Garbowski et al. [28,29].

As it was mentioned above, the sidewalls of the box are often weakened by cutouts for various purposes. There can be several reasons for this: tab-like cutouts, ventilation holes/openings (mainly for agricultural products), viewing windows, etc. These solutions have a negative influence on the compression strength of the boxes, and this phenomenon was investigated by several authors [8,30,31,32,33,34,35,36].

In 2020, Garbowski et al. [35] indicated that a smaller hole on the sidewall would ensure better (greater) compression strength, but it is necessary that it would be located at the center of the wall. On the other hand, the McKee formula cannot give precise results in the aspect of a cutout independent of its position, shape, and size. An additional important issue in the compression strength investigation is the length-to-width ratio of the CCB. Research has shown that if the aspect ratio changes from 1 to 3, then the compression strength increases at first and then decreases; furthermore the maximum compression force can be observed when the aspect ratio was approximately 1.6 [37,38]. 

It must be mentioned here that after a careful literature review, the authors could not find any published research that measures and/or analyzes the interconnection of these variables on such a wide range of box dimensions and large and growing cutout sizes with a primary focus on material reduction. There is a gap in the literature for a wide variety of experiments in this area. The papers published so far mainly focused on the mechanical properties of individual/special box dimensions, with only a partial understanding of the overall relationships. This study attempts to develop an estimation method for the compression strength of cardboard boxes with various (growing) cutout sizes. Cutout technology is generally used for various purposes such as ventilation, reduction in material, viewing windows, etc. First, empirical box compression tests were performed on a wide range of dimensions, using 250 box samples in total to observe the changes in mechanical strength, and then an analysis was performed comparing the empirical results with the analytical results of the McKee formula. Finally, this paper presents an estimation map for the McKee constant with a given number of cardboard samples along various cutouts. Therefore, this paper can provide novel insights into circumstances for packaging engineers to design boxes based on experimental data using a simple method for compression strength estimation.

## 2. Materials and Methods

### 2.1. Samples

**Figure 1:**
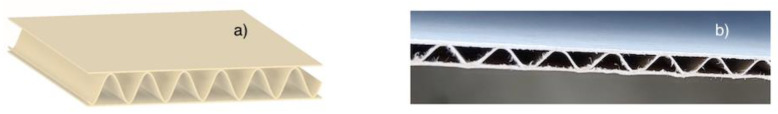
Structure (**a**) and cross-section (**b**) of corrugated cardboard sample used for this study.

For this study, single-wall B-flute corrugated cardboard (Figure 1) boxes with different cutouts were used that were made from the same cardboard material quality. Table 1 shows the mechanical specifications for the tested corrugated cardboard. The corrugated cardboard material composition contained the following:

- Outer liner: 210 GD2 (weight 210 g/m^2^, coated white lined chipboard with grey back, quality class 2);
- Fluting medium: 120 HC (weight 120 g/m^2^, high compression Wellenstoff);
- Inner liner: 130 TL 3 (weight 130 g/m^2^, Testliner, quality class 3).

**Figure 2:**
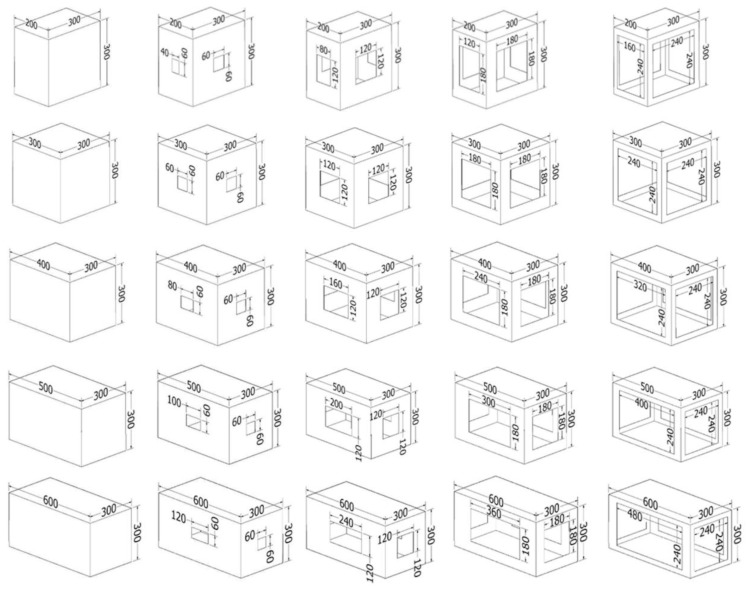
Five types of cutouts used on samples with different dimensions.

Both the assembly of the boxes and the gluing process were carried out by hand. For this study, 5 boxes with different lengths and 5 different cutout areas were used. Table 2 and Figure 2 show the configurations for the samples. Each sample had the same width and height of 300 mm. The ratios of the cutout areas to the sidewall were the following: 0%, 4%, 16%, 36%, and 64%, respectively. The cutouts were cut from the center of the sidewalls along all four sides. In order to evaluate the measurement results, 10 samples were tested for each cutout group for each dimension, for a total of 250 samples.

### 2.2. Measurement Setup 

**Figure 3:**
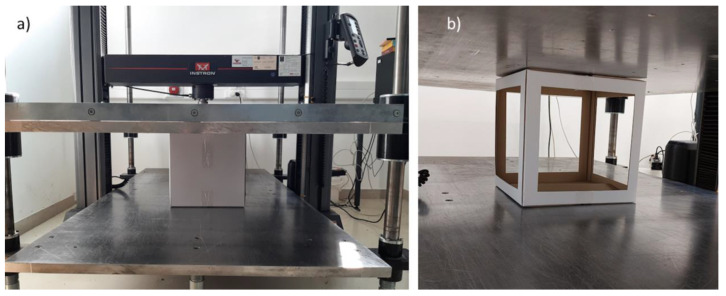
Box compression test (BCT): (**a**) control sample and (**b**) cutout sample (64% cutout area).

To observe and determine the maximum compression force, a BCT (box compression test) was performed on each box. This is a simple load test between a stationary and a moving steel plate. The device used for BCT measurements can be seen in Figure 3. During the tests, the compression force and deformation were continuously recorded. Before the test series, the samples were preconditioned at 30 °C ± 1 °C and 20–30% RH (relative humidity) for 24 h and then conditioned at 23 °C ± 1 °C and 50 ± 2% RH for 24 h in a climate-testing chamber in accordance with the ASTM D4332 standard [39]. Then, BCT tests were promptly executed after conditioning to avoid any additional hygroscopic phenomenon. BCTs were performed according to the ASTM D642 standard [40], so the testing speed of the crosshead was 12.7 mm/min ± 2.5 mm/min until the failure of the box occurred. 

### 2.3. Box Compression Strength—McKee’s Formula

The empirical BCT values for various box dimensions (Table 2) were then compared with the BCT values calculated by the McKee formula, so each variant case was compared with the values given by the McKee formula in order to determine which of the variable parameters of the McKee formula could be modified for a unique dimension. The formula, which was created in 1963 by McKee, has already been modified several times since its establishment, and currently, it is widely used in two variants. One version is the full McKee formula (Equation (1)), which is mainly used by researchers and developers and is relatively too complicated for everyday use [20,41]:

(1) $$P=kP_{m}^{b}{\left(\sqrt{D_{x}D_{y}}\right)}^{\left(1-b\right)}Z^{\left(2b-1\right)}$$

The above formula (Equation (1)) gives the BCT value of the box in terms of the compressive force and is obtained using the following corrugated cardboard box parameters: *P_m_* represents the edgewise compression strength of the combined board, and Z is the perimeter of the box. In addition to these two basic parameters, the in-machine flexural stiffness of the combined board and the cross-machine flexural stiffness of the combined board are denoted by *D_x_* and *D_y_*. The latter must be taken into account since the compressive force on the box causes the sidewalls to bulge, which can occur both in the in-machine and cross-machine directions. These values are considered to be non-variable factors for a given box type and size. The context includes the empirical constants denoted by *k* and *b* of which *k* is the multiplier of the whole equation, while *b*, the empirical constant, is in the exponent, and therefore, the choice of their values will have a major influence on the result obtained for the BCT value. It should be specifically noted here that while the constant is a multiplier without a unit of measure, the constant also modifies the unit of measure in the equation.

The original McKee formula suggests a *k* constant of 2.028 and a *b* constant of 0.746. With these data, Equation (1) takes the following form [42]:

(2) $$P=2.028P_{m}^{0.746}{\left(\sqrt{D_{x}D_{y}}\right)}^{0.254}Z^{0.492}$$

In practice, it is preferable to use a simplified version of the above formula, which is given by Equation (3) [42]:

(3) $$P=kP_{m}\left(\sqrt{hZ}\right)$$

The simplified McKee formula uses the cardboard thickness in the equation instead of the bending stiffness, which is denoted by *h*. The creator of the equation assumes that bending stiffness varies proportionally with the thickness of the cardboard, and although this is a significant mechanical simplification, feedback from the industry confirms this assumption in general practice [43].

In comparing the BCT results, the simplified McKee formula was used, and the aim was to determine whether the practical value of the equation was appropriate or whether another equation should be chosen for the CCB with sidewall cutouts. For our study, the manufacturer gave a value of 5.3 for the material and box type used, and this was used in the simplified calculation. The manufacturer did not reveal the original source of the *k* value’s calculation.

### 2.4. Data Analysis

The characteristics of the measured datasets can be described with statistical indicators, including the maximum, minimum, average, standard deviation, and standard error for each group. The statistical models were determined using the linear regression method because it was the best fit for the empirical data. With this method, a simple function (y(*x*) = a*x* + b) was calculated. This method was used in each group where the cutout areas were the predictor variables, and the compression forces were the output values. In linear regression analysis, the R^2^ (coefficient of determination) values were used to determine the accuracy of the statistical models. The range of R^2^ was between 0 and 1. If the R^2^ value equaled one, then this indicated that the model can predict the dependent variable (in our case, the compression force) with 100% accuracy. In order to determine the differences between different the groups, a one-way analysis of variance (ANOVA) was executed with the Tukey post hoc test. The significance level was determined at *p* < 0.05 for the statistical analysis. The following software programs were used for the statistical evaluations: MATLAB R2021b (MathWorks Inc., Natick, MA, USA) and JASP 0.16.3 (the University of Amsterdam, The Netherlands).

## 3. Results and Discussion

**Figure 4:**
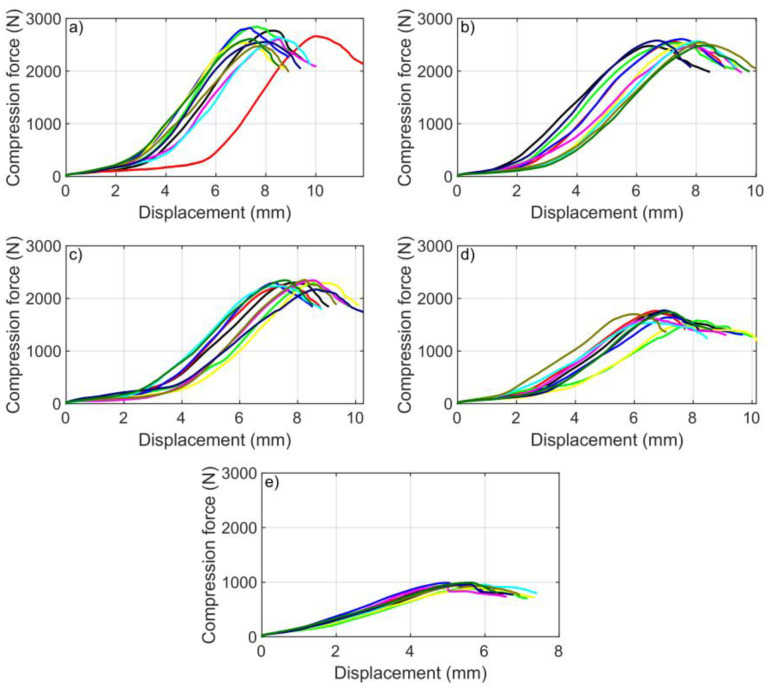
BCT curves: (**a**) with 0% cutout, (**b**) 4% cutout, (**c**) 16% cutout, (**d**) 36% cutout, and (**e**) with 64% cutout (10 samples for box size of 400 × 300 × 300 mm).

The force–displacement diagrams of the BCT measurements were drawn for all box variants. As a sample, for the boxes of 400 mm × 300 mm × 300 mm, five diagrams are presented for the cut and uncut samples with the ten measurements of each (Figure 4). Figure 4a shows compression force–displacement functions for the 0% cutout, Figure 4b for the 4% cutout, Figure 4c for the 16% cutout, Figure 4d for the 36% cutout, and Figure 4e for the 64% cutout. The numerical data for these measurements are also given in Table 3. For each additional box type, all the datasets are available, which can be requested from the authors. 

**Figure 5:**
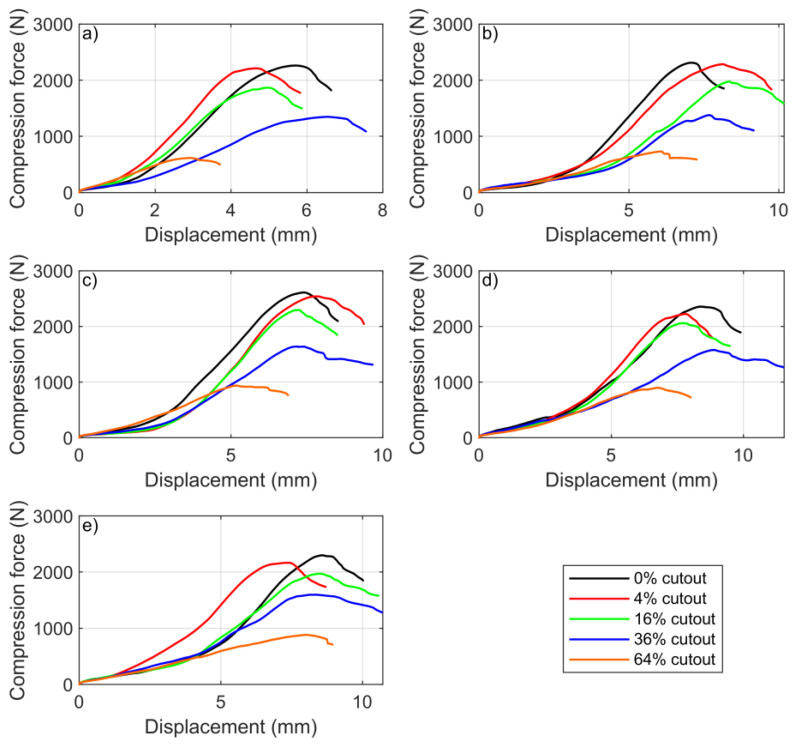
BCT quasi-median values: (**a**) 600 × 300 × 300 mm box, (**b**) 500 × 300 × 300 mm box, (**c**) 400 × 300 × 300 mm box, (**d**) 300 × 300 × 300 mm box, and (**e**) 200 × 300 × 300 mm box.

Table 4 contains the minimum, maximum, mean, fifth highest BCT value (which can be considered as quasi-median), and standard deviation of the BCT results for the different box types, and Figure 5 shows the compression force–displacement diagrams for all box types for all cutout ratios. The diagrams show the fifth highest (quasi-median) maximum compression force value of the ten measurements. By comparing the data in the fifth and sixth columns, it is clear that the quasi-median value was a good approximation of the sample mean, and since these were real measured data, it was reasonable to plot this value. The last column of Table 4 shows the magnitude of the standard deviation of the samples. The 400 mm box size was considered ideal in many respects. The highest measured BCT value was for this box type (2843 N). For box lengths of 500 and 600 mm, the standard deviation increased for cutout areas above 16%. For 500 mm box lengths, it increased from 59 N (36%) to 85 N (64%), and for 600 mm box lengths, it increased from 59 N (16%) to 87 N (36%).

A cutout of 64% had extremely low values for all box types. The box behaved almost as an edge protector, not capable of bearing the load, even though in principle, the compressive strength of the box is determined by the vertical edges bent into an L shape. Despite this, the fact that the compressive strength of the box was radically reduced demonstrates that the total surface area of the sidewalls played a significant role in the absorption of compressive forces. 

### 3.1. Linear Regression

**Figure 6:**
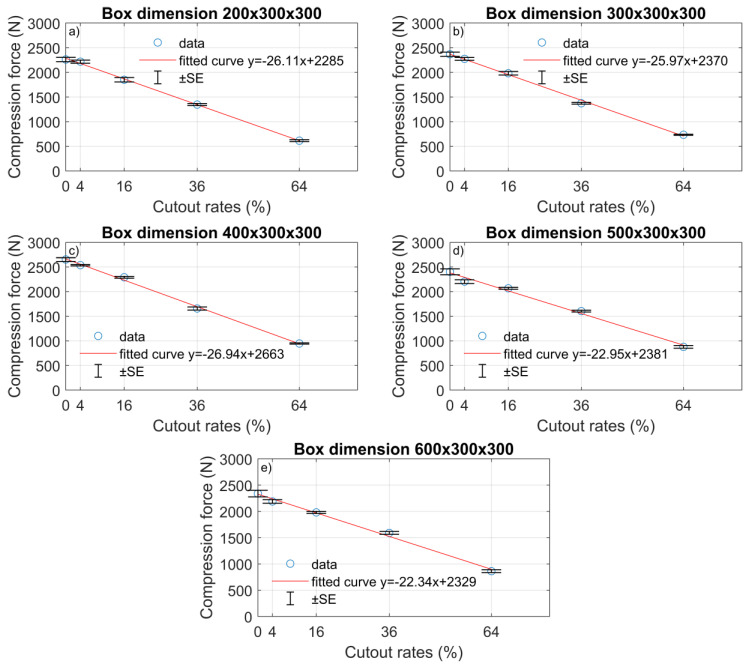
Averages of maximum compression forces and its standard errors (SE): (**a**) 200 × 300 × 300 mm box, (**b**) 300 × 300 × 300 mm box, (**c**) 400 × 300 × 300 mm box (**d**) 500 × 300 × 300 mm box, and (**e**) 600 × 300 × 300 mm box.

Using descriptive statistics, the average maximum compression force values were determined in each group (shown in Table 4). Figure 6 shows these average values and the standard errors related to the cutout areas. Based on the data points (Figure 6), it can be identified that there was a decreasing tendency in the average maximum compression forces, as in other studies [8,31,35]. The statistical models were determined in the five box design groups using linear regression. These fitted lines can be seen in Figure 6. There were very high R2 values in each case, between 0.9904 and 0.9988 (Table 5). These indicate that the models described the measured data with very high accuracy. Using these linear functions, the compression forces could be predicted in each box dimension group separately if the box was made from single-wall B-flute corrugated cardboard. However, it has to be noted that in the model of 500 mm and 600 mm groups, there were residuals, so if the cutout rate increased up to 100%, there were residual compression force values that were not possible. The 400 × 300 × 300 group had the steepest regression slope and the highest y-intercept.

**Figure 7:**
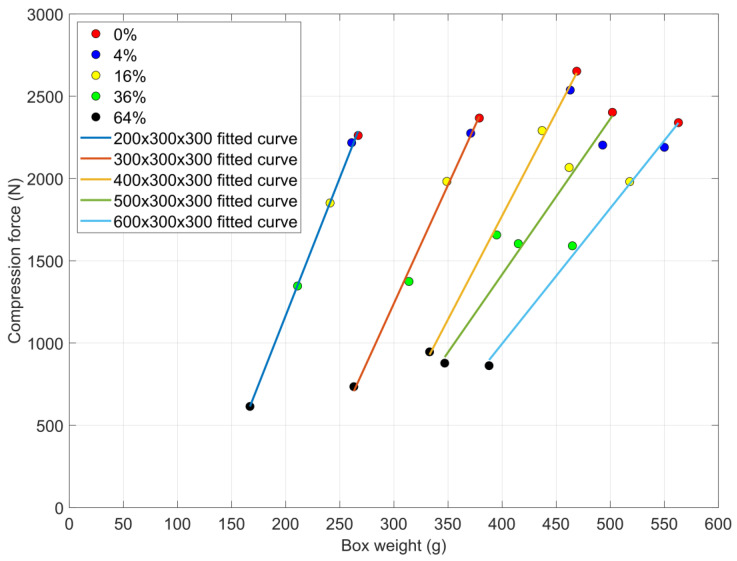
Box weights and averages of maximum compression forces diagram along the various cutouts.

Figure 7 shows the box weight–average maximum compression force diagrams. Data points with different colors represent the results of different cutout areas. The lines with different colors are the fitted curves of the data points that represent the different box sizes. The data points on the yellow line are above all other measured data, so the 400 × 300 × 300 sized boxes had the best average compression force results. This result shows a good correlation with a previous study [37], where the authors showed that the compressive strength increased at first and then decreased, and in [37], the maximum compression strength appeared when the length-to-width ratio was about 1.6. In our study, the optimal length-to-width ratio was 1.33. It should be highlighted that in [37], the material of the tested corrugated box was different (BC flute corrugated cardboard with five layers). In this study, the highest average compression force was 2651 N ± 39 N for the 400 × 300 × 300 box with 0% cutout area. The lowest average compression force was 614 N ±19 N in the 200 × 300 × 300 box size group with 64% cutout area. In almost each cutout area group (except the 4%), the 200 × 300 × 300 boxes were shown to have the least stiff behavior. In the 4% cutout area group, the 600 × 300 × 300 box had the minimum average compression force (Table 4). The 64% cutout group produced the lowest average compression forces in each box size group. This kind of weight reduction significantly reduced the compressive strength of the boxes. Figure 7 also shows that the slopes of the fitted curves decrease when the size of the box increases. That means the cutouts had more impact related to the weight reduction when the size of the box was bigger.

### 3.2. McKee Comparison

**Figure 8:**
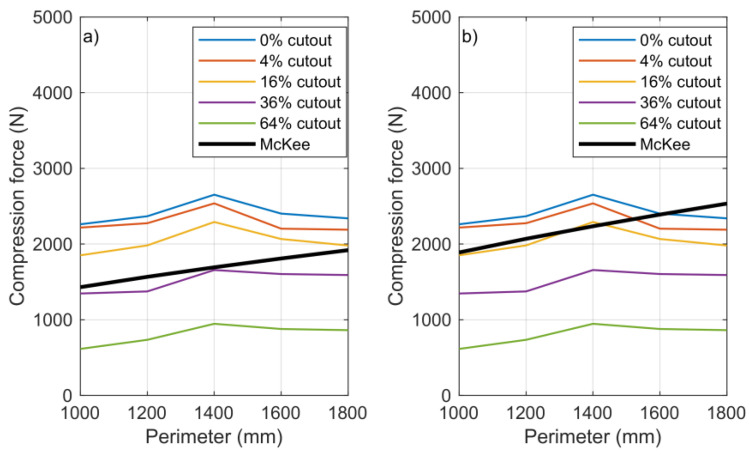
Summary data of BCT values calculated from the McKee equation and measured average BCT values for different cutout rates: (**a**) *k* = 5.3 and (**b**) *k* = 7.

**Figure 9:**
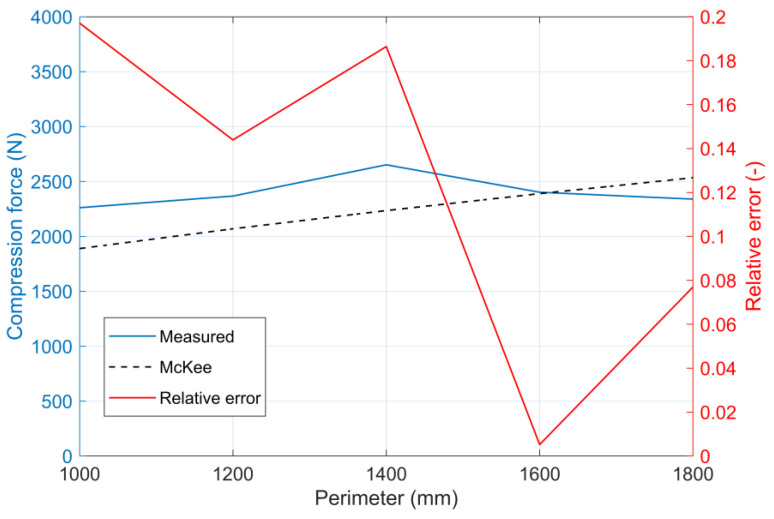
The relative error of the BCT results obtained with the simplified McKee formula (*k* = 7) for the uncut versions of each box type.

The BCT value, calculated with a coefficient of 5.3 given by the manufacturer, was significantly lower than the measured BCT values for all uncut boxes. In order to calculate the BCT values using the simplified McKee equation more consistently with the measured BCT values, a first approximation of the coefficient *k* of 7 was chosen. The correlation of the BCT values calculated with the coefficient *k* = 5.3 with the measured BCT values is shown in Figure 8a. The relation of the BCT values calculated with coefficient *k* = 7 to the measured BCT values is shown in Figure 8b. Figure 9 shows the relative error calculated from the results for the different sizes of uncut boxes.

From the diagram, it appears that there was rather an optimum value (1400 mm perimeter), below and above which the measured BCT value decreased. However, in both the simplified and the full McKee context, the box perimeter is a multiplier, and hence, as the perimeter increases, the BCT values should also increase. Practically, it can be seen that an unlimited increase in the perimeter simply could not result in a linear increase in the BCT value. 

**Figure 10:**
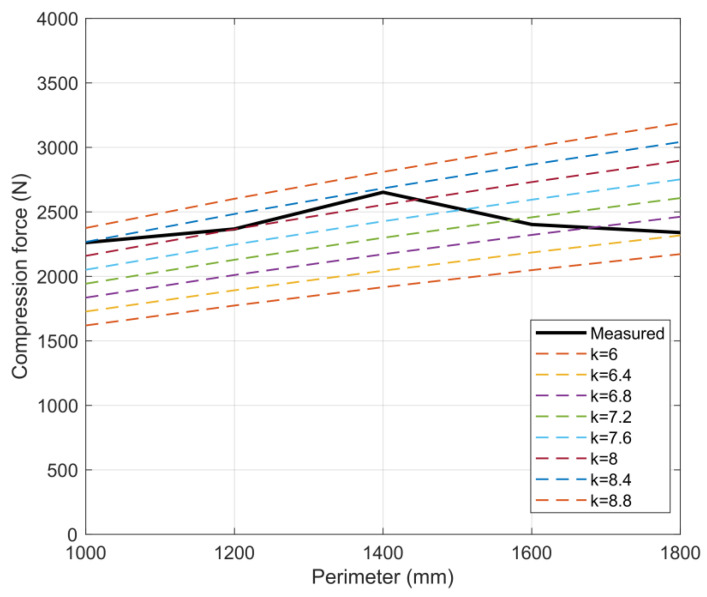
Iteration approximation of coefficient *k* for averages of BCT test results for uncut boxes of different sizes.

As shown in Figure 10, the value of factor *k* varied between 6 and 8.8 based on the perimeter linear growth of the simplified McKee relation, with a step size of 0.4. The averages of the measured BCT values for each box variation are shown in this interval. From the diagram, the coefficients *k*, applicable to each box size included in the measurement, can be assigned to that box size. This also implies that applying the same *k* coefficient to boxes of different geometric sizes would lead to an error in the estimation of the BCT value. For the cutout samples, the discrepancies were even more significant and could not be handled using the simplified McKee formula.

In a similar study, Garbowski et al. [35] calculated BCT values using the full McKee formula and compared them with their measured results. In their study, the authors performed the calculations and measurements for several different types of corrugated cardboard, but the geometric dimensions of the box were in a relatively narrow range, only for 300 × 200 × 200 and 300 × 200 × 300 mm (length × width × height). Garbowski et al. calculated the expected BCT value with a relative error of 15.5% for their samples.

In our own measurements, the geometric size of the boxes varied over a wider range, but for the 400 mm boxes with the best BCT results, the error of the simplified McKee formula was extremely high (around 18%), as the measured BCT value was significantly higher than the theoretical value calculated with the simplified McKee formula. For boxes with a length of 500 mm, the measured value and the value obtained from the McKee formula were almost coincident, and the error rate was below 1%. For box lengths of 600 mm, the BCT value given by the McKee relation fell below the measured BCT value; here, the error was almost 8%. The measured BCT values up to a box length of 500 mm exceeded the BCT values calculated from the McKee function for uncut boxes everywhere. It is particularly noticeable that for boxes of 400 mm in length, which gave the highest BCT values in the measurements, the McKee correlation led to much lower BCT values. The linear relationship between the perimeter and the BCT value in the simple McKee formula seems to be falsified based on our actual measurements. This suggests that the McKee relationship is limited as the box perimeter increases, presumably due to the change in the length–width ratio. 

### 3.3. Limitations for Practice

1. The experimental method in this study used only the B-flute corrugated cardboard, so the results fell within a narrow range. In reality, boxes made of corrugated cardboard have an extremely wide range and variation, such as A, B, C, and other flutes with different numbers of layers, etc.; therefore, the results of this study may be limited for general use, but they do cover an important issue for a possible reduction in material and packaging engineering design.
2. It should also be noted here that there are some environmental circumstances that significantly affect box compression strength, including the changes in temperature and relative humidity and the difference between dynamic and static load. This study did not observe these conditions.

## 4. Conclusions

- The boxes with cutout areas of 0%, 4%, 16%, 36%, and 64% showed a linear decreasing tendency in compression force. The linear regression model described the measured data with very high accuracy.
- The 400 × 300 × 300 mm sized boxes showed the best average compression force results when the length-to-width ratio was 1.33.
- The 64% cutout group produced the lowest average compression forces in each box size group, which means the 64% cutout significantly reduced the compressive strength of the boxes.
- For cutout corrugated cardboard boxes, the discrepancies in mechanical strength are more significant and cannot be handled with the simplified McKee formula. In some cases, the error of the simplified McKee formula was extremely high.
- The McKee relationship is limited as the box perimeter increases, presumably due to the change in the length–width ratio.
- An optimum perimeter value (1400 mm) could be found, below and above which the measured BCT value decreased, as opposed to the McKee formula in which the perimeter is a multiplier.
- The McKee formula works with relatively high error on corrugated cardboard boxes with sidewall cutouts and cannot follow the tendency of compressive forces along various perimeters.

## Figures and Tables

**Table 1:** Mechanical specification of the B-flute corrugated cardboard tested.

Properties	Specification	Applied Standard
Board Thickness	2.8 mm (±10%)	ISO 3034 (FEFCO No.3)
Grammage	512 g/m^2^ (±10%)	ISO 536:1995
Edge crush test (ECT)	5.1 kN/m (±15%)	ISO 3037 (FEFCO No.8)
Bursting strength (BST)	676 kPa (±15%)	ISO 2759 (FEFCO No.4)

**Table 2:** Configuration for dimensions of samples for this study (same width and height of 300 mm).

Length (mm)	Perimeter (mm)	Area without Top and Bottom Flaps (mm^2^)	Sizes of Cutout (mm)	Cutout Area (mm^2^)	Cutout Ratio (%)
200	1000	300,000	40 × 60/60 × 60 80 × 120/120 × 120 120 × 180/180 × 180 160 × 240/240 × 240	0 12,000 48,000 108,000 192,000	0 4 16 36 64
300	1200	360,000	60 × 60/60 × 60 120 × 120/120 × 120 180 × 180/180 × 180 240 × 240/240 × 240	0 14,400 57,600 129,600 230,400	0 4 16 36 64
400	1400	420,000	80 × 60/60 × 60 160 × 120/120 × 120 240 × 180/180 × 180 320 × 240/240 × 240	0 16,800 67,200 151,200 268,800	0 4 16 36 64
500	1600	480,000	100 × 60/60 × 60 200 × 120/120 × 120 300 × 180/180 × 180 400 × 240/240 × 240	0 19,200 76,800 172,800 307,200	0 4 16 36 64
600	1800	540,000	120 × 60/60 × 60 240 × 120/120 × 120 360 × 180/180 × 180 480 × 240/240 × 240	0 21,600 86,400 194,400 345,600	0 4 16 36 64

**Table 3:** BCT (N) values of 10 measurements for different cutouts (400 × 300 × 300 mm).

Sample Number	Compression Force (N) for Different Cutout Areas				
	0%	4%	16%	36%	64%
1	2769	2479	2299	1729	937
2	2665	2566	2257	1762	962
3	2843	2528	2295	1581	879
4	2819	2608	2295	1638	988
5	2532	2539	2324	1486	899
6	2606	2465	2342	1616	916
7	2644	2571	2235	1543	993
8	2477	2547	2352	1699	935
9	2555	2582	2170	1772	962
10	2610	2487	2344	1741	992

**Table 4:** BCT results for different box dimensions.

Length (mm)	Cutout Rates (%)	Min. Force (N)	Max. Force (N)	Mean Force (N)	Quasi-Median (N)	Standard Deviation (N)
200	0	2026	2462	2261	2261	146
	4	2092	2364	2218	2211	101
	16	1607	2037	1851	1866	145
	36	1245	1448	1347	1348	64
	64	460	686	615	616	62
300	0	2189	2595	2367	2311	136
	4	2117	2382	2275	2284	91
	16	1841	2198	1982	1973	114
	36	1296	1470	1374	1377	54
	64	690	815	735	731	33
400	0	2477	2843	2652	2610	123
	4	2465	2608	2537	2539	47
	16	2170	2352	2291	2295	57
	36	1486	1772	1657	1638	99
	64	879	993	946	937	40
500	0	2040	2667	2402	2356	189
	4	1945	2316	2203	2224	119
	16	1964	2159	2067	2058	63
	36	1541	1714	1604	1577	59
	64	748	1004	878	899	85
600	0	2053	2624	2339	2300	198
	4	2006	2371	2190	2167	108
	16	1880	2082	1981	1972	59
	36	1476	1745	1591	1598	87
	64	627	915	862	882	85

**Table 5:** Statistical model of the BCT values for sample boxes.

Length (mm)	Fitted Linear Curve	R^2^
200	−26.11*x* + 2285	0.9988
300	−25.97*x* + 2370	0.9972
400	−26.94*x* + 2663	0.9974
500	−22.95*x* + 2381	0.9904
600	−22.34*x* + 2329	0.994

## Data Availability

The data published in this research are available on request from the first author and corresponding authors.
